# *Euphorbia supina* Extracts Block NADPH Oxidase-Mediated, Ceramide-Induced Apoptosis Initiated by Diesel Particulate Matter

**DOI:** 10.3390/ph15040431

**Published:** 2022-03-31

**Authors:** Kyong-Oh Shin, Sungeun Kim, Bokyung Kim, Hye-Yoon Park, Eunhee Jung, Garyun Kim, Donghee Kim, Hwang Eui Cho, Yoshikazu Uchida, Kyungho Park

**Affiliations:** 1 Department of Food Science & Nutrition, and Convergence Program of Material Science for Medicine and Pharmaceutics, Hallym University, Chuncheon 24252, Korea; 0194768809@hanmail.net (K.-O.S.); sungeuna27@naver.com (S.K.); bokyung0725@naver.com (B.K.); 2 The Korean Institute of Nutrition, Hallym University, Chuncheon 24252, Korea; 3 LaSS Lipid Institute (LLI), LaSS Inc., Chuncheon 24252, Korea; 4 Biological and Genetic Resources Assessment Division, National Institute of Biological Resources, Incheon 22689, Korea; rejoice077@me.go.kr (H.-Y.P.); ehjung@korea.kr (E.J.); ryun31@korea.kr (G.K.); 5 College of Pharmacy, Chonbuk National University, Jeonju 54896, Korea; ideal1022@naver.com (D.K.); hecho@jbnu.ac.kr (H.E.C.)

**Keywords:** apoptosis, ceramide, diesel particulate matters, *Euphorbia supina* Rafin, nicotinamide adenine dinucleotide phosphate oxidase, sphingomyelinase

## Abstract

Air pollutants contribute to the development of diseases such as asthma, chronic obstructive pulmonary disease (COPD), pulmonary cancer, cardiovascular problems, and some skin diseases. We recently found that a major air pollutant, diesel particulate matter (DPM), induces apoptosis in human keratinocytes by increasing a proapoptotic lipid mediator, ceramide. DPM activates nicotinamide adenine dinucleotide phosphate (NADPH) oxidase (NOX), which stimulates sphingomyelinase, leading to an increased conversion of sphingomyelin to ceramide. Interestingly, we characterized that although NOX is a reactive oxygen species (ROS) generator, the activation of sphingomyelinases by NOX is an ROS-independent mechanism. A Korean weed, prostrate spurge *Euphorbia supina* Rafin (ESR), has been used for centuries as a folk medicine to treat bronchitis, hepatitis, hemorrhage, and skin inflammation. Flavonoids, terpenes and tannins are enriched in ESR, and although ESR has proven antioxidative activity, its biological activities are largely unknown. Here, we investigate whether and how ESR protects keratinocytes against DPM-mediated apoptosis. We found that ESR-extracts (ESR-Ex) protect keratinocytes from DPM-induced apoptosis by inhibiting NOX activation in keratinocytes in response to DPM. We also demonstrated that ESR-Ex suppresses NOX activation via a blockage of the aryl hydrocarbon receptor (AhR) activation-mediated transcription of neutrophil cytosolic factor 1 (NCF1)/p47phox, a subunit of NOX. Our study reveals previously uncharacterized biological activity of ESR-Ex; i.e., its inhibition of Ahr and NOX activation. Thus, because the inhibition of NOX has already been developed to treat NOX-mediated diseases, including various types of cardiovascular diseases and cancers, initiated by air pollutants and because AhR activation contributes to the development of chronic inflammatory diseases, our study provides further advantages for the medical use of ESR.

## 1. Introduction

Air pollutants are one of the main environmental factors in the development of diseases such as asthma, chronic obstructive pulmonary disease (COPD), pulmonary cancer, cardiovascular problems, some skin diseases (contact hypersensitivity and dermatitis), the impairment of skin barrier function and the acceleration of premature skin aging. Diesel particulate matter (DPM) is produced from diesel engines, is comprised of polycyclic aromatic hydrocarbons, benzene, formaldehyde, acetaldehyde, and 1,3-butadiene, and is a major air pollutant.

Nicotinamide adenine dinucleotide phosphate (NADPH) oxidases (NOXs) are plasma membrane-bound enzymes that produce reactive oxygen species (ROS) [1]. NOX is activated in response to stimuli, such as cytokines, angiotensin, or ultraviolet irradiation [1]. Because NOXs have been found to contribute to the development of cardiovascular diseases, NOX inhibitors have already been developed [2,3]. Pertinent to our study, DPM stimulates ROS production through NOX activation [4,5].

Our prior study demonstrated that DPM induces apoptosis in human keratinocytes (KC)(HaCat cells) via a pro-apoptotic lipid mediator, ceramide-dependent pathway. DPM activates NOX followed by sphingomyelinase activation, resulting in increased ceramide production via the hydrolysis of sphingomyelin, leading to ceramide-inducing apoptosis [6]. Interestingly, although NOX is a ROS generator, its activation of sphingomyelinases occurs via an ROS-independent mechanism [6].

A weed, the Korean prostrate spurge *Euphorbia supina* Rafin (ESR), has been used as a folk medicine to treat several types of diseases, including bronchitis, hepatitis, hemorrhage, and skin inflammation [7]. ESR extracts (ESR-Ex) contain flavonids, terpenes and tannins [7] and exhibit anti-oxidative activities [7]. Several biological activities of ESR, such as suppressing manganese-induced neurotoxicity [8] and melanogenesis [9] and inducing apoptosis in U937 human leukemia cells [10] have already been characterized. Yet, its biological activities are still largely unknown.

Here, we investigated whether and how ESR-Ex protects keratinocytes against DPM-mediated apoptosis in cultured human KC, and we discovered that ESR-Ex inhibits NOX activation via the blockage of Aryl hydrocarbon receptor (Ahr) activation-mediated increases in the transcription of neutrophil cytosolic factor 1 (NCF1)/p47phox, a subunit of NOX in KC in response to DPM exposure, resulting in suppressed DPM-mediated ceramide-inducing apoptosis.

## 2. Results

### 2.1. Component of ESR Extracts

An LC-MS/MS analysis identified that ESR extracts (70% ethanol) (ESR-Ex) contain several flavonoids and tannins, such as quercetin, kaempferol, isorhamnetin, gallic acid and their derivatives (Table 1).

### 2.2. ESR-Ex Suppresses DPM-Induced Decreases in Cell Viability and NOX Activation

**Figure 1:**
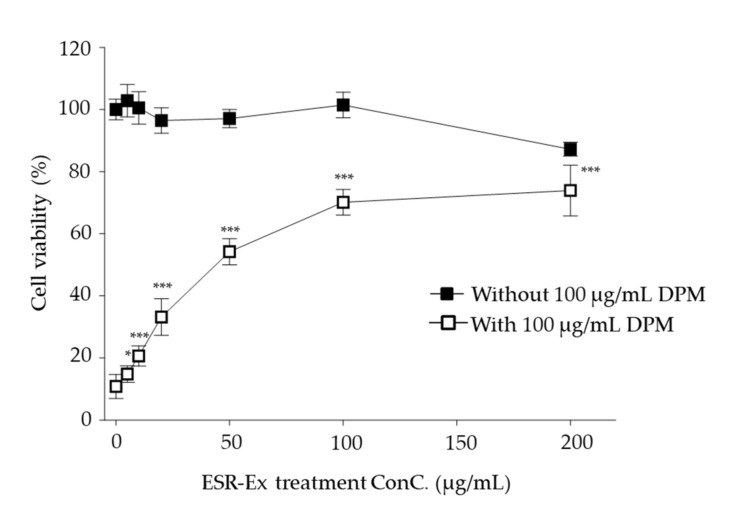
ESR-Ex suppresses decreases in cell viability in KC treated with DPM. KCs were incubated with indicated concentration of ESR-Ex or vehicle for 30 min, and then further cultured with 100 µg/mL of DPM for 24 h. Cell viability was measured by water-soluble tetrazolium salt cell quantification method. All values are mean ± SD (*n* = 3). Statistically significant differences of ESR + DPM (open square), *** *p* < 0.001 or * *p* < 0.05 vs. vehicle control without DPM. See details in Materials and Methods Section.

We first assessed that influence of ESR on cell viability and then investigated whether ESR-Ex suppresses decreases in cell viability in KC exposed to DPM. Up to 100 g/mL ESR alone does not affect cell viability, while 200 µg/mL modestly decreases cell viability (Figure 1). Consistent with our prior study [6], DPM reduced cell viability in KC (not shown), while ESR-Ex significantly suppressed decreased cell viability in KC treated with DPM in a dose-dependent fashion (Figure 1). We used 100 µg/mL of ESR in the following studies.

**Figure 2:**
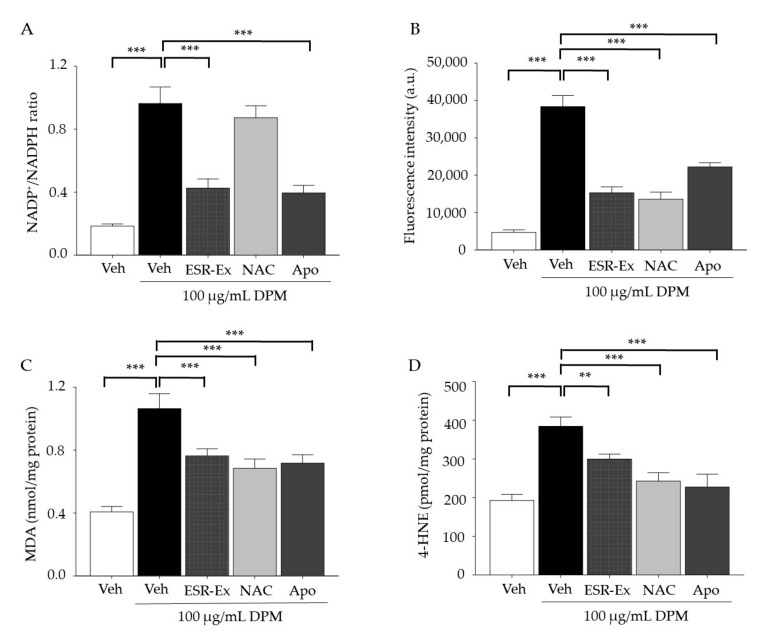
ESR-Ex suppresses both NOX activation and increases in ROS production in KC treated with DPM. KC were incubated with or without ESR-Ex (100 μg/mL), NAC (1 mM), Apo (100 μM) or vehicle for 30 min, and then further cultured with 100 µg/mL of DPM for 24 h. Measuring the ratio of NADP+ to NADPH with LC-ESI-MS/MS (API 3200 QTRAP mass, AB/SCIEX) by multiple reaction monitoring mode (MRM) (**A**). Intercellular ROS production was determined by fluorospectrophotometer (**B**). Malondialdehyde (MDA) (**C**) and 4-hydroxynonenal (4-HNE) (**D**) were determined by LC-ESI-MS/MS after dansyl hydrazine derivatization. All values are mean ± SD (*n* = 3). *** *p* < 0.001 or ** *p* < 0.01 vs. indicated group. See details in Materials and Methods Section.

We next examined the effect of ESR-Ex on NOX activation by DPM. Consistent with our prior study [6], DPM activated NOX in KC, while its activation was significantly suppressed by ESR-Ex, as well as a specific pharmacological inhibitor of NOX, apocynin (Apo) (Figure 2A). However, as expected, an established antioxidant N-acetyl cysteine (NAC) did not inhibit NOX activation (Figure 2A).

We next measured cellular ROS levels by a DCFDA agent. ROS production was significantly increased in KC incubated with DPM, while the elevation of cellular ROS was significantly suppressed by the coincubation of DPM with ESR-Ex, Apo or NAC (Figure 2B). We further confirmed ROS production by assaying malondialdehyde and 4-hydroxynoneal production. Malondialdehyde and 4-hydroxynoneal generation were found to be significantly decreased in KC co-incubation with DPM and ESR-Ex, Apo or NAC (Figure 2C,D). These results suggest that ESR-Ex has the ability to: (1) suppress decreases in cell viability; (2) inhibit NOX activation; (3) suppress ROS production in KC following DPM treatment.

### 2.3. ESR-Ex Suppresses Changes in Ceramide Profiles and Cell Toxicity in KC Exposed to DPM

**Figure 3:**
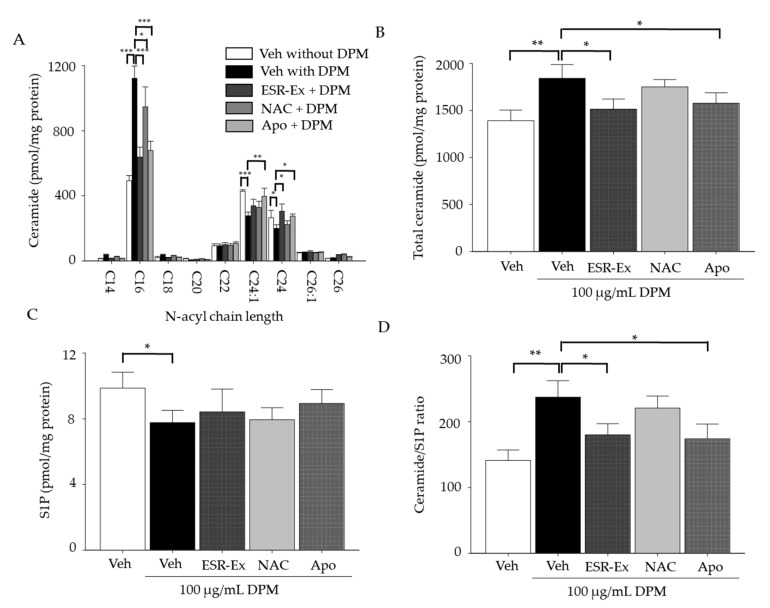
ESR-Ex suppresses increases in total ceramide production and alteration of ceramide molecular profiles and normalizes the ratio of ceramide to S1P in KC exposed to DPM. KC were incubated with or without ESR-Ex (100 μg/mL), NAC (1 mM), Apo (100 μM) or vehicle for 30 min, and then further cultured with 100 µg/mL of DPM for 24 h. Total ceramide (**A**), ceramide species of different amide-linked fatty acid (**B**), sphingosine-1-phosphate (**C**), Ceramide/S1P ratio (**D**) were measured by LC-ESI-MS/MS. All values are mean ± SD (*n* = 3). *** *p* < 0.001, ** *p* < 0.01, or * *p* < 0.05 vs. indicated group. See details in Materials and Methods Section.

Since our prior study shows that an increases in a pro-apoptotic lipid mediator, ceramide, increases apoptosis in KC following exposure to DPM [6], we next investigated the effect of ESR-Ex on ceramide levels in KC treated with DPM. Total ceramide levels were significantly increased in KC treated with DPM, while increases in ceramide were significantly diminished in KC co-incubation of DPM with ESR-Ex or Apo, but not NAC (Figure 3A). Further analysis of ceramide molecular species revealed ceramide containing shorter amide-linked fatty acid (C16) (short chain ceramide) species, which has a proapoptotic feature among ceramide species [11], were significantly increased and conversely, ceramides containing longer amide-linked fatty acid (C24:1 and 24:0) (long chain ceramide) were significantly decreased in KC following DPM treatment (Figure 3B). ESR-Ex and Apo, but not NAC, suppressed increases in short chain ceramide and decreases in long-chain ceramide production in KC treated with DPM.

**Figure 4:**
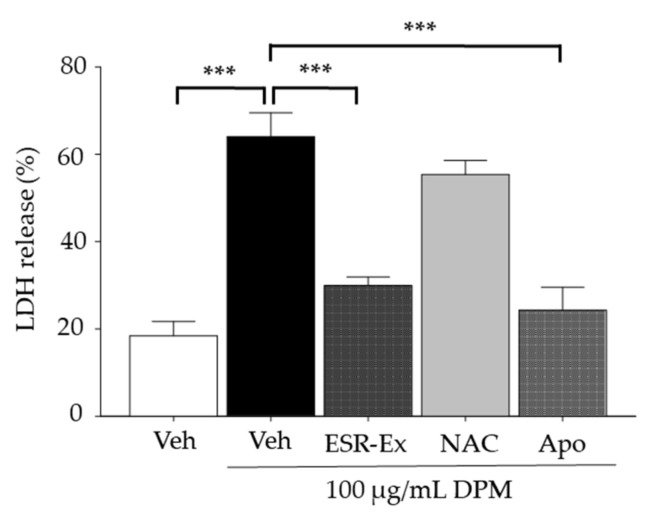
ESR-Ex decreases cell toxicity in KC treated with DPM. KC were incubated with or without ESR-Ex (100 μg/mL), NAC (1 mM), Apo (100 μM) or vehicle for 30 min, and then further cultured with 100 µg/mL of DPM for 24 h. Cell toxicity was assessed by lactic dehydrogenase releasing assay. All values are mean ± SD (*n* = 3). *** *p* < 0.001 vs. indicated group. See details in Materials and Methods Section.

Excess ceramide is pro-apoptotic, while the metabolic conversion of ceramide to sphingosine-1-phosphate (S1P) (via ceramide hydrolysis by ceramidase followed by phosphorylation by sphingosine kinase) rescues cells from apoptosis [12]. We found that DPM decreases S1P levels in KC following DPM treatment, resulting in an increased ratio of ceramide to S1P (Figure 3C,D). ESR-Ex and Apo, but not NAC, modestly reversed these decreases in S1P. Along with the suppression of increases in ceramide production, ESR-Ex and Apo, but not NAC, attenuated an increased ratio of ceramide to S1P in KC exposed to DPM. We further demonstrated that both ESR-Ex and Apo, but not NAC, significantly reduced cell toxicity (assessed by lactic dehydrogenase release from cells) in KC treated with DPM (Figure 4).

These results suggest that ESR-Ex and Apo suppress an increase in proapoptotic short chain ceramide production and maintain a metabolic rescue mechanism (ceramide-to-S1P), thereby reducing cell toxicity in KC following DPM. Moreover, although NAC inhibits ROS production (Figure 2C,D), NAC does not suppress increases in bulk- and short chain ceramide or decreases in long chain ceramide production in KC treated with DPM. Thus, changes in ceramide synthesis should be a result of the activation of NOX, but not ROS generated by NOX.

### 2.4. ESR-Ex Suppresses DPM-Mediated Activation of Sphingomyelinases

**Figure 5:**
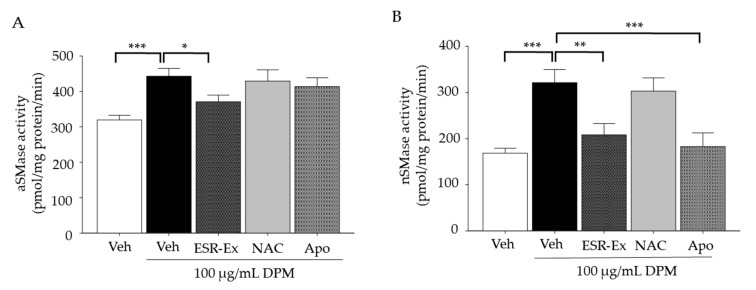
ESR-Ex suppresses DPM activation of sphingomyelinases. KC were incubated with or without ESR-Ex (100 μg/mL), NAC (1 mM), Apo (100 μM) or vehicle for 30 min, and then further cultured with 100 µg/mL of DPM for 24 h. Activities of acidic sphingomyelinase (**A**) and neutral sphingomyelinase (**B**) were measured by LC-ESI-MS/MS. All values are mean ± SD (*n* = 3). *** *p* < 0.001, ** *p* < 0.01, or * *p* < 0.05 vs. indicated group. See details in Materials and Methods Section.

We demonstrated that sphingomyelinase is activated by NOX activation and results in increased ceramide production by sphingomyelin hydrolysis [6]. We next assessed the effects of ESR-Ex on sphingomyelinase activation in KC exposed to DPM. DPM activated both acidic and neutral sphingomyelinases in KC, while both ESR-Ex and Apo significantly suppressed the activation of neutral sphingomyelinases in KC treated with DPM (Figure 5A,B). ESR-Ex also suppresses increases in acidic sphingomyelinase activity in cells exposed to DPM (Figure 5A). However, NAC did not suppress either sphingomyelinase activation. These results suggest that ESR-Ex blocks the activation of sphingomyelinases. Moreover, although NAC inhibits ROS production in KC following DPM exposure (Figure 2C,D), sphingomyelinase activation is not inhibited by NAC, confirming our prior findings; i.e., sphingomyelinase is activated by NOX, but not by ROS generated by NOX [6].

### 2.5. ESR-Ex Suppresses NOX Activation via Blockage of AhR-Induced p47phox Production

DPM contains chemicals such as 2,3,7,8-tetrachlorodibenzo-*p*-dioxin and benzo(a)pyrene that activate AhR [13,14], leading to NOX activation via increases in the transcription of the NADPH oxidase subunit, neutrophil cytosolic factor 1 (NCF1)/p47phox [15,16]. Because some polyphenols contained in ESR-Ex, including kaempferol and quercetin, inhibit AhR activation [17,18] (Table 1), we hypothesize that ESR-Ex suppresses p47phox production via the inhibition of AhR activation.

**Figure 6:**
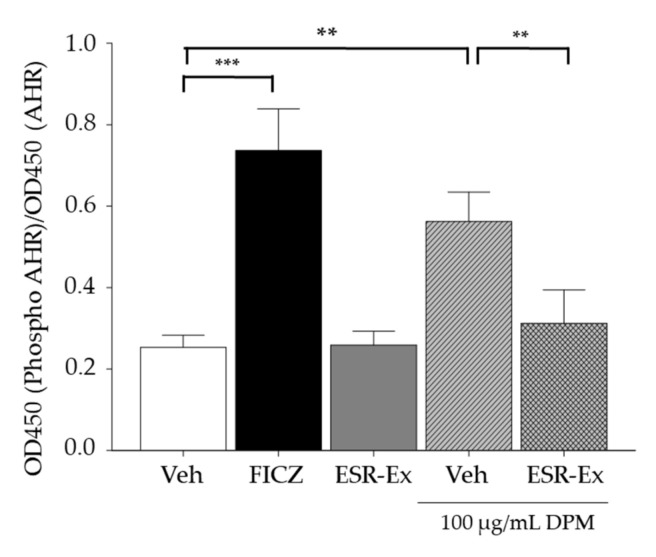
ESR-Ex suppresses phosphorylation of AhR in KC exposed to DPM. KC were incubated with or without FITZ (AhR ligand), ESR-Ex (100 μg/mL) or vehicle for 30 min, and then further cultured with 100 µg/mL of DPM for 24 h. Ratio of phosphorylated AhR to Ahr was measured by AHR Cell-Based Phosphorylation ELISA. All values are mean ± SD (*n* = 3). *** *p* < 0.001 or ** *p* < 0.01 vs. indicated group. See details in Materials and Methods Section.

We first investigated the activation of AhR in KC exposed to DPM (assessed by AhR phosphorylation). We confirmed AhR activation in our cultured system using an established AhR ligand, FICZ [19] (Figure 6). We then demonstrated the activation of Ahr in KC following DPM exposure, while DPM-mediated AhR activation was suppressed in cells by coincubation with ESR.

**Figure 7:**
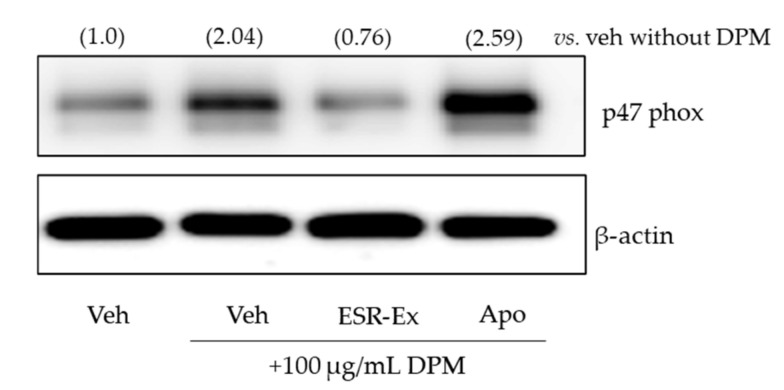
ESR-Ex suppresses increases in p47phox in KC exposed to DPM. KC were incubated with or without ESR-Ex (100 μg/mL), Apo (100 μM) or vehicle for 30 min, and then further cultured with 100 µg/mL of DPM for 24 h. p47phox and β-actin were analyzed by Western blot. Fold changes compared with the vehicle without DPM are shown in parentheses. See details in Materials and Methods Section.

We next investigated levels of p47phox protein of which production is transcriptionally regulated by AhR [19]. Western blot analysis showed that DPM increases p47phox protein levels in KCs and that ESR-Ex suppressed these increases of p47phox in cells (Figure 6). Because Apo inhibits NOX activation by binding to subunit proteins of NOX, it did not affect p47phox production. Thus Apo did not suppress p47phox protein production (Figure 7). These results suggest that ESR-Ex inhibits NOX activation via a blockage of DMP-induced AhR activation-mediated p47phox protein synthesis.

### 2.6. ESR-Ex Suppresses DPM-Induced Apoptosis

Finally, we found that ESR-Ex suppresses DPM-induced apoptosis. ESR-Ex diminished DPM-induced increases in the pro-apoptotic regulator, BAX and an inducer of apoptosis, caspase 3, and conversely decreased anti-apoptotic regulator Bcl-2 expression in KC (Figure 7). These results suggest that ESR-Ex protects KC against DPM-induced apoptosis.

## 3. Discussion

**Figure 8:**
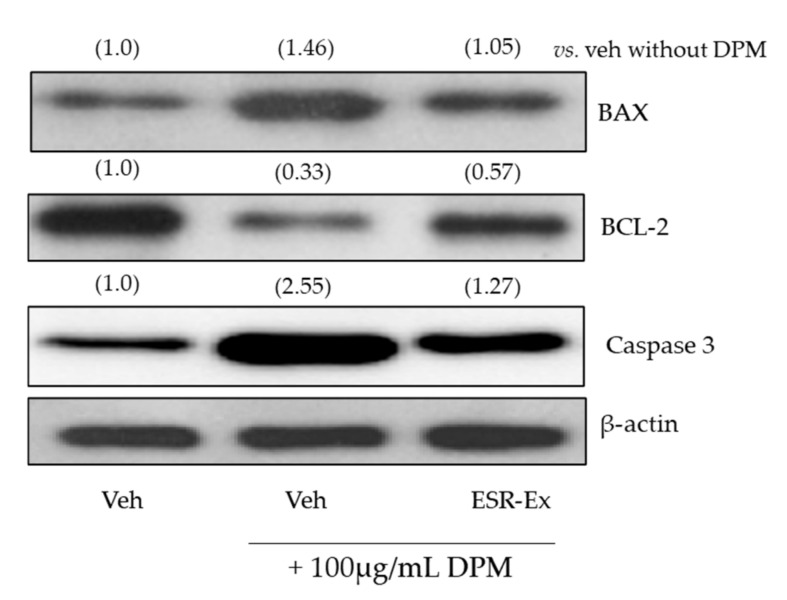
ESR-Ex suppresses increases in BAX, Caspase 3, and conversely decreases in BCL-2 proteins levels in KC exposed to DPM. KC were incubated with or without ESR-Ex (100 μg/mL), Apo (100 μM) or vehicle for 30 min, and then further cultured with 100 µg/mL of DPM for 24 h. BAX, BLC-2, Caspase 3 and β-actin were analyzed by Western blot. Fold changes compared with the vehicle without DPM are shown in parentheses. See details in Materials and Methods Section.

We investigated the ability of ESR-Ex to protect KC from DPM (a major air pollutant)-induced apoptosis. We also characterized that ESR-Ex inhibits NOX activation by DPM, thereby blocking ceramide-induced apoptosis via the suppression of NOX-dependent sphingomyelinase activation (Figure 8).

How does ESR-Ex inhibit DPM-mediated NOX activation? As described above in the Results section (*2.5. ESR-Ex suppresses NOX activation* via *blockage of AhR-induced p47phox production*), chemicals contained in DPM increase the transcription of p47phox via AhR activation [13,14,15,16]. Kaempferol and quercetin, contained in ESR-Ex (Table 1), inhibit AhR activation [17,18]. Hence, we suggest that ESR-Ex inhibits the DPM-induced activation of NOX by suppressing p47phox (a subunit of NOX) production.

Sphingomyelinase is activated in response to stimuli, such as inflammatory cytokines and ROS [20,21]. NOX is an ROS generator. However, our prior and current studies suggest that DPM increases sphingomyelinase activity in KC via a NOX-dependent, but not by ROS, which is generated by a NOX-independent pathway. So how does NOX activate sphingomyelinase? Acidic sphingomyelinase can be recruited from cytosol to plasma in response to stimuli [3]. Both acidic sphingomyelinase recruited to plasma membranes by DPM exposure and plasma membrane-residential neutral sphingomyelinase may be activated via interaction with NOX on a plasma membrane. However, the mechanism of NOX-dependent activation of both neutral and acidic sphingomyelinases remains unclear.

(1) DPM activates AhR followed by increased transactivation of p47phox (a subunit of NOX required for NOX activation); (2) Increases in p47 activate NOX; (3) NOX activates sphingomyelinase by a ROS-independent (unidentified) mechanism, leading to increases in ceramide production by sphingomyelin hydrolysis; and (4) Increased ceramide induces apoptosis in KC.

ESR-Ex has been used as a folk medicine for centuries, and its anti-oxidative activities are well known. Here, we have shown previously unrealized biological activities of ESR-Ex; i.e., as an AhR inhibitor and a NOX inhibitor. Our study could lead to new clinical indications for ESR-Ex; i.e., its use in prevention of DPM-mediated diseases, including asthma, chronic obstructive pulmonary disease (COPD), pulmonary cancer, and cardiovascular diseases initiated by environmental air pollutants and cigarette smoke. Since air pollutants are involved in accelerating premature skin aging [22,23]. ESR-Ex also could be useful as a skin care agent. NOX inhibitors already are used to treat cardiovascular diseases [2,3]. Additionally, AhR is involved in the development of some chronic inflammatory diseases, such as inflammatory bowel diseases (IBD) and cancer development [24,25], so ESR-Ex may also help to treat AhR-mediated and NOX-mediated (via AhR activation) diseases.

**Figure 9:**
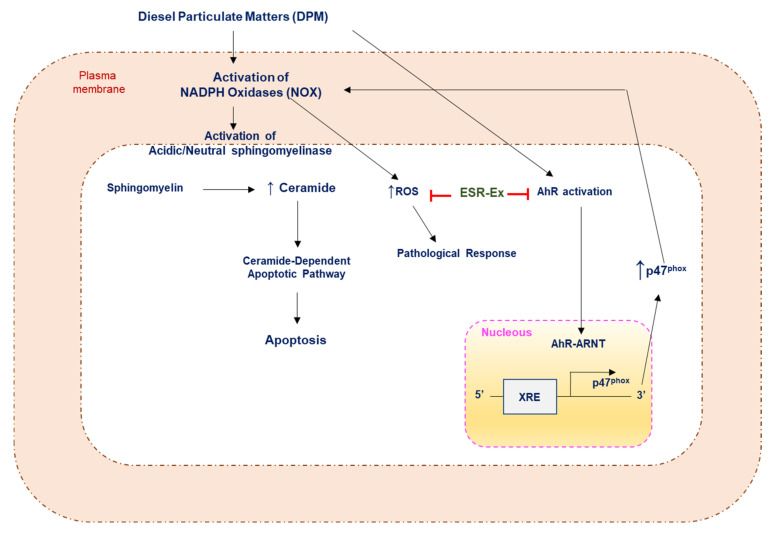
Proposed mechanism of ESR-Ex intervention in DPM-mediated ceramide-induced apoptosis.

In conclusion, ESR-Ex protects KC from DPM-induced apoptosis by inhibiting AhR activation-mediated NOX activation, followed by a blockage of increases in an apoptotic lipid mediator (ceramide production) by activating sphingomyelinase and sphingomyelin hydrolysis (Figure 9).

## 4. Materials and Methods

### 4.1. Materials

Diesel particulate extract used in the present study is Standard Reference Material 1975 (SRM 1975), purchased from the National Institute of Standards and Technology (NIST) (Gaithersburg, MD, USA). Nicotinamide, NADPH, NADP, and 2, 7-dichlorofluorescin diacetate were obtained from Sigma-Aldrich (St. Louis, MO, USA). Apo, N-acetylcystine. C17-S1P, S1P (d18:1), sphingosine, ceramides (fatty acid lengths C8, C12, C16, C18, C22, C24, and C24:1), C17-ceramide (d17:1/C18:0), and C12-sphingomyeline sphingosine, ceramides (fatty acid lengths C12, C16, C18, C22, C24, and C24:1) were obtained from Avanti Polar Lipids (Alabaster, AL, USA). Organic solvents for sphingolipid extraction or LC-MS/MS analysis were purchased from Merck (Darmstadt, Germany).

### 4.2. ESR-Ex Preparation

ESR was provided by the National Institute of Biological Resources (Incheon, Korea). The dry stem and leaf of ESR-Ex (200 g) were extracted with 70% ethanol reflux for 90 min at 70 °C three times. The extracts were filtered with Whatman NO.3 filter paper (Maids one, England) and concentrated by a vacuum evaporator (Rotavapor R-200; Buchi, Flawil, Switzerland). Subsequently, the concentrated extract was freeze-dried (Hanil Co. Ltd., Gimpo, South Korea) to obtain a dry powder sample (ESR-Ex) with a yield of about 18% (36 g).

### 4.3. Identification of Phenolic Acid and Flavonoids in ESR-Ex

The extracts were separated using a ExionLC™ Series UHPLC system (Shimazu, Tokyo, Japan). Furthermore, chromatographic separation was performed on a Waters ACQUITY UPLC HSS T3 C18 column (2.1 × 50 mm, 1.8 μm) and a thermos tatted at 40 °C was used and then pre-equilibrated in solvent A (0.1% formic acid in water). Extracts were eluted with increasing percentages of solvent B (acetonitrile with 0.1% formic acid) at a flow rate of 0.5 mL/min. The elution gradient steps were as follows: 0–1 min, 0% B; 1–4 min, gradient to 70% B; 4–6 min, 100% B; 6–6.2 min, gradient to 0% B; 6.2–10 min, 0% B. The injection volume was 5 μL and the effluent was connected to an ESI-triple quadrupole-linear ion trap (QTRAP)-Mass spectrometry (Applied Biosystems 5500 QTRAP, Applied Biosystems, Foster City, CA, USA).

Mass detection was performed in an API5500 QTRAP equipped with an electrospray ionization (ESI) source and a triple quadrupole-ion trap spectrometer controlled by the Analyst 1.7.1 software. The negative ionization mode was applied, and the optimized instrument settings were as follows: curtain gas (CUR): 30.0 psi; collision gas (CAD): medium; ion spray (IS) voltage: −4500 V; source temperature: 500 °C; GS1: 60 psi and GS2: 50 psi; The mass detector was programmed to perform two consecutive modes: enhanced mass scan (EMS) and multiple reaction monitoring–information-dependent acquisition–triggering enhanced product ion (MRM-IDA-EPI) mode analysis. EMS was employed to obtain full scan spectra, so as to provide an overview of all the ions in the sample. The settings used were: declustering potential (DP) −120 V, entrance potential (EP) −9.5 V, collision energy (CE) −30 V. Spectra were recorded in negative ion mode between *m*/*z* 200 and 1000. Phenolic acids and flavonoids in ESR-Ex extracts were identified by comparing the retention times and mass spectra with the reference standards.

### 4.4. Cell Culture

Immortalized, non-transformed (HaCaT) human keratinocytes (KC), derived from human epidermis (a gift from N. Fusenig, Heidelberg, Germany), were grown, as described previously [26,27]. Briefly, HaCaT KC were maintained in Dulbecco’s modified Eagle’s medium (DMEM) containing 10% fetal calf serum (FCS) and 1% penicillin/streptomycin (P/S). Similar to our prior studies [26,27], culture medium was switched to serum-free, antibiotics-free KC growth medium (154 CF, Thermo Fisher Scientific, Waltham, MA, USA) containing 0.07 mM calcium chloride and growth supplements (Thermo Fisher Scientific) 1 day prior to DPM treatment.

### 4.5. Cell Viability and Cytotoxicity

Cell viability was measured by the water-soluble tetrazolium salt (WST) method using the cell counting kit-8 (CCK-8) assay kit (Dojindo, Kumamoto, Japan) in accordance with the manufacturer’s instruction. Cytotoxicity was assayed using in vitro Toxicology assay kit, lactic dehydrogenase based (Sigma-Aldrich).

### 4.6. NADPH Oxidases

Activity of NADPH oxidase cell membrane fractions was measured by the lucigenin chemiluminescence assay kit using N, N′-Dimethyl-9,9′-bicridium dinitrate and 1 μM NADPH (Sigma-Aldrich), in accordance with the manufacturer’s instruction.

Activity of NADPH oxidases was measured by the ratio of NADP+ to NADPH using LC-ESI-MS/MS (API 3200 QTRAP mass, AB/SCIEX, Framingham, MA, USA) by multiple reaction monitoring (MRM) mode, as described previously [6]

### 4.7. Detection of Cellular ROS

To detect the production of cellular reactive oxygen species (ROS), including superoxide (O_2_^−^) and hydrogen peroxide (H_2_O_2_), the oxidant-sensing probe 2,7-dichlorodihydrofluorescein diacetate (DCFH-DA) (abcam, Cambridge, MA, USA) was used, as described previously [28]. Briefly, ROS production was analyzed using a fluorescence microscopy (Eclipse Ti-U; Nikon Corporation, Tokyo, Japan) and fluorospectrophotometer (Molecular devices M2e, Molecular Devices, Sunnyvale, CA, USA) with 485 nm of excitation and 520 nm of emission filters and was expressed as fluorescence intensity (a.u.).

### 4.8. Measurement of Ceramide, and Sphingosine-1-Phosphate (S1P)

Cells were treated with or without NOX inhibitor (Apocynin [APO], 100 μM) or ROS generation (N-Acetylcysteine [NAC], 1 mM) for 30 min and then further incubated with DPM for 24 h, followed by extraction of lipids as we reported previously [26,27]. Ceramide, and S1P were quantitated using LC-ESI-MS/MS (API 3200 QTRAP mass), as we described previously [26,27]. The ceramide MS/MS transitions (*m*/*z*) were 510→264 for C14-ceramide, 538→264 for C16-ceramide, 552→264 for C17-ceramide, 566→264 for C18-ceramide, 594→264 for C20-ceramide, 648→264 for C24:1-ceramide, 650→264 for C24-ceramide, 676→264 for C26:1-ceramide, and 678→264 for C26-ceramide, respectively. The S1P MS/MS transitions (*m*/*z*) were 366→250 for C17 S1P as an internal standard and 380→264 for C18 sphingosine 1-phosphate, respectively. Data were acquired using Analyst 1.5.1 software (Applied Biosystems). Sphingolipid levels are expressed as pmol per mg protein.

### 4.9. Sphingomyelinase Assay

Activities of acidic or neutral sphingomyelinase were measured as described previously [6]. Briefly, cells suspended in assay buffers (acidic sphingomyelinase: 250 mM sodium-acetate, 0.2% Triton X-100, pH 4.5, and neutral sphingomyelinase: 20 mM HEPES, 0.2% Triton X-100, pH 7.4) were incubated with 5 nmol of C12-sphingomyeline for 20 min at 37 °C. The reaction was stopped by the addition of CHCl_3_: CH_3_OH (2:1, *v*/*v*). The organic phases were dried and were resolved in MeOH, and then analyzed by LC-MS/MS. The activities of both sphingomyelinase are expressed as pmol (C12-ceramide) per mg protein per min.

### 4.10. Western Blot Analysis

Protein levels of BAX, BCL-2, caspase 3 and β-actin were assessed using Western blot analysis, as described previously [26,27]. Briefly, cell lysates, prepared in radioimmunoprecipitation assay buffer, were resolved by electrophoresis on 4–12% Bis-Tris protein gel (Invitrogen). Resultant bands were blotted onto polyvinylidene difluoride membranes, probed with anti-BAX, anti- BCL-2, anti-caspase 3(Santa Cruz Biotechnology, Dallas, TX, USA), and anti-human β-actin (Sigma-Aldrich, St. Louis, MO, USA), and detected using enhanced chemiluminescence (Thermo Fisher Scientific). Band intensity was measured with a LAS-3000 (Fujifilm, Tokyo, Japan).

### 4.11. AhR Phosphorylation Assay

AhR activation was assessed by AhR phosphorylation using Phospho-AHR Cell-Based Phosphorylation ELISA (LSBio, Seattle, WA, USA) in accordance with the manufacturer’s instructions. A pharmacological ligand of AhR FICZ was purchased from Sigma-Aldrich.

### 4.12. Statistical Analyses

Results were expressed as the means ± standard deviation (SD). Statistical analyses were performed using the GraphPad Prism 8.0.1 software (GraphPad Software, San Diego, CA, USA). Significance between groups was determined by a one-way analysis of variance (ANOVA) coupled with Dunnett’s multiple comparison test. The *p* values were set at either *** <0.001, ** <0.01, or * <0.05.

## Figures and Tables

**Table 1:** Identification of flavonoids and tannins in *Euphorbia supina* Raf Extracts (ESR-Ex) (LC-MS Analysis).

Compound	[M-H]^−^	Mass Fragmentation
Quercetin	301	301, 273, 179, 153
Quercetin 3-hexoside	463	463, 301, 300,283, 271, 255, 179, 151
Quercetin-3-O-(2G-α-L-rhamnosyl)-rutinoside	755	755, 300, 283, 271, 255, 179, 151
Kaempferol	287	287, 258, 165, 153, 121
Kaempferol 3-hexoside	447	447, 285, 255
Kaempferol-3-O-rutinoside	593	593, 285, 255, 227, 151
Isorhamnetin	316	316, 301, 272, 256, 164, 151
Isorhamnetin-3-O-neohesperidoside	623	623, 459, 314, 299, 285, 271, 257
Gallic acid	169	169, 125, 97
Gallic acid glucoside	331	169, 125

## Data Availability

Data is contained within the article.

## References

[1] Bedard K., Krause K.H. (2007). The NOX family of ROS-generating NADPH oxidases: Physiology and pathophysiology. Physiol. Rev..

[2] Elbatreek M.H., Pachado M.P., Cuadrado A., Jandeleit-Dahm K., Schmidt H. (2019). Reactive Oxygen Comes of Age: Mechanism-Based Therapy of Diabetic End-Organ Damage. Trends Endocrinol. Metab..

[3] Li C., Wang A., Wu Y., Gulbins E., Grassme H., Zhao Z. (2019). Acid Sphingomyelinase-Ceramide System in Bacterial Infections. Cell Physiol. Biochem..

[4] Chen J.H., Chou F.P., Lin H.H., Wang C.J. (2005). Gaseous nitrogen oxide repressed benzo[a]pyrene-induced human lung fibroblast cell apoptosis via inhibiting JNK1 signals. Arch. Toxicol..

[5] Tarafdar A., Pula G. (2018). The Role of NADPH Oxidases and Oxidative Stress in Neurodegenerative Disorders. Int. J. Mol. Sci..

[6] Lee H.S., Park H.Y., Kwon S.P., Kim B., Lee Y., Kim S., Shin K.O., Park K. (2020). NADPH Oxidase-Mediated Activation of Neutral Sphingomyelinase Is Responsible for Diesel Particulate Extract-Induced Keratinocyte Apoptosis. Int. J. Mol. Sci..

[7] Song Y., Jeong S.W., Lee W.S., Park S., Kim Y.H., Kim G.S., Lee S.J., Jin J.S., Kim C.Y., Lee J.E. (2014). Determination of Polyphenol Components of Korean Prostrate Spurge (Euphorbia supina) by Using Liquid Chromatography-Tandem Mass Spectrometry: Overall Contribution to Antioxidant Activity. J. Anal. Methods Chem..

[8] Bahar E., Lee G.H., Bhattarai K.R., Lee H.Y., Choi M.K., Rashid H.O., Kim J.Y., Chae H.J., Yoon H. (2017). Polyphenolic Extract of Euphorbia supina Attenuates Manganese-Induced Neurotoxicity by Enhancing Antioxidant Activity through Regulation of ER Stress and ER Stress-Mediated Apoptosis. Int. J. Mol. Sci..

[9] Kang S.H., Jeon Y.D., Cha J.Y., Hwang S.W., Lee H.Y., Park M., Lee B.R., Shin M.K., Kim S.J., Shin S.M. (2018). Antioxidant and skin-whitening effects of aerial part of Euphorbia supina Raf. Extract. BMC Complement. Altern. Med..

[10] Han M.H., Lee W.S., Nagappan A., Kim H.J., Park C., Kim G.Y., Hong S.H., Kim N.D., Kim G., Ryu C.H. (2016). Polyphenols from Korean prostrate spurge Euphorbia supina induce apoptosis through the Fas-associated extrinsic pathway and activation of ERK in human leukemic U937 cells. Oncol. Rep..

[11] Wattenberg B.W. (2018). The long and the short of ceramides. J. Biol. Chem..

[12] Uchida Y., Houben E., Park K., Douangpanya S., Lee Y.M., Wu B.X., Hannun Y.A., Radin N.S., Elias P.M., Holleran W.M. (2010). Hydrolytic pathway protects against ceramide-induced apoptosis in keratinocytes exposed to UVB. J. Investig. Dermatol..

[13] Brinchmann B.C., Skuland T., Rambol M.H., Szoke K., Brinchmann J.E., Gutleb A.C., Moschini E., Kubatova A., Kukowski K., Le Ferrec E. (2018). Lipophilic components of diesel exhaust particles induce pro-inflammatory responses in human endothelial cells through AhR dependent pathway(s). Part Fibre Toxicol..

[14] Palkova L., Vondracek J., Trilecova L., Ciganek M., Pencikova K., Neca J., Milcova A., Topinka J., Machala M. (2015). The aryl hydrocarbon receptor-mediated and genotoxic effects of fractionated extract of standard reference diesel exhaust particle material in pulmonary, liver and prostate cells. Toxicol. In Vitro.

[15] Koizumi M., Tatebe J., Watanabe I., Yamazaki J., Ikeda T., Morita T. (2014). Aryl hydrocarbon receptor mediates indoxyl sulfate-induced cellular senescence in human umbilical vein endothelial cells. J. Atheroscler. Thromb..

[16] Pinel-Marie M.L., Sparfel L., Desmots S., Fardel O. (2009). Aryl hydrocarbon receptor-dependent induction of the NADPH oxidase subunit NCF1/p47 phox expression leading to priming of human macrophage oxidative burst. Free Radic. Biol. Med..

[17] Macpherson L., Matthews J. (2010). Inhibition of aryl hydrocarbon receptor-dependent transcription by resveratrol or kaempferol is independent of estrogen receptor alpha expression in human breast cancer cells. Cancer Lett..

[18] Vrba J., Kren V., Vacek J., Papouskova B., Ulrichova J. (2012). Quercetin, quercetin glycosides and taxifolin differ in their ability to induce AhR activation and CYP1A1 expression in HepG2 cells. Phytother. Res..

[19] Tanaka M., Fujikawa M., Oguro A., Itoh K., Vogel C.F.A., Ishihara Y. (2021). Involvement of the Microglial Aryl Hydrocarbon Receptor in Neuroinflammation and Vasogenic Edema after Ischemic Stroke. Cells.

[20] Jana A., Pahan K. (2004). Fibrillar amyloid-beta peptides kill human primary neurons via NADPH oxidase-mediated activation of neutral sphingomyelinase. Implications for Alzheimer’s disease. J. Biol. Chem..

[21] Reiss L.K., Raffetseder U., Gibbert L., Drescher H.K., Streetz K.L., Schwarz A., Martin C., Uhlig S., Adam D. (2020). Reevaluation of Lung Injury in TNF-Induced Shock: The Role of the Acid Sphingomyelinase. Mediat. Inflamm..

[22] Schikowski T., Huls A. (2020). Air Pollution and Skin Aging. Curr. Environ. Health Rep..

[23] Shin K.O., Uchida Y., Park K. (2022). Diesel Particulate Extract Accelerates Premature Skin Aging in human Fibroblasts via Ceramide-1-Phosphate-Mediated Signaling Pathway. Int. J. Med. Sci..

[24] Pernomian L., Duarte-Silva M., de Barros Cardoso C.R. (2020). The Aryl Hydrocarbon Receptor (AHR) as a Potential Target for the Control of Intestinal Inflammation: Insights from an Immune and Bacteria Sensor Receptor. Clin. Rev. Allergy Immunol..

[25] Leclerc D., Staats Pires A.C., Guillemin G.J., Gilot D. (2021). Detrimental activation of AhR pathway in cancer: An overview of therapeutic strategies. Curr. Opin. Immunol..

[26] Park K., Elias P.M., Shin K.O., Lee Y.M., Hupe M., Borkowski A.W., Gallo R.L., Saba J., Holleran W.M., Uchida Y. (2013). A novel role of a lipid species, sphingosine-1-phosphate, in epithelial innate immunity. Mol. Cell. Biol..

[27] Park K., Ikushiro H., Seo H.S., Shin K.O., Kim Y.I., Kim J.Y., Lee Y.M., Yano T., Holleran W.M., Elias P. (2016). ER stress stimulates production of the key antimicrobial peptide, cathelicidin, by forming a previously unidentified intracellular S1P signaling complex. Proc. Natl. Acad. Sci. USA.

[28] Rosenkranz A.R., Schmaldienst S., Stuhlmeier K.M., Chen W., Knapp W., Zlabinger G.J. (1992). A microplate assay for the detection of oxidative products using 2’,7’-dichlorofluorescin-diacetate. J. Immunol. Methods.

